# Photocatalytic Hydrogen Production Performance of ZnCdS/CoWO_4_ Heterojunctions in the Reforming of Lignin Model Compounds

**DOI:** 10.3390/ma18184401

**Published:** 2025-09-21

**Authors:** Jianxu Zhang, Jingwei Li, Weisheng Guan

**Affiliations:** 1 School of Water and Environment, Chang’an University, Xi’an 710064, China; 2 Key Laboratory of Subsurface Hydrology and Ecological Effect in Arid Region of the Ministry of Education, Chang’an University, Xi’an 710064, China; 3 Key Laboratory of Eco-Hydrology and Water Security in Arid and Semi-Arid Regions of Ministry of Water Resources, Chang’an University, Xi’an 710064, China

**Keywords:** photocatalytic, photo-reforming, hydrogen generation, heterojunction

## Abstract

Biomass reforming under mild conditions for synergistic hydrogen production, driven by renewable solar energy, has rapidly emerged as a promising strategy that not only enables the efficient reutilization of biomass but also facilitates the generation of high-purity hydrogen. In this work, ZnCdS (ZCS) nanoparticles and CoWO_4_ (CW) nanocrystals were assembled via a solvothermal method to construct a ZCS/CW S-type heterojunction composite. The resultant materials’ physicochemical characteristics were methodically described. With lignin model compounds (PP-ol) and sodium lignosulfonate as substrates, the ZnCdS/CoWO_4_-10% catalyst demonstrated a significant generation of hydrogen activity, producing hydrogen at rates of 223.30 μmol·g^−1^·h^−1^ and 140.28 μmol·g^−1^·h^−1^, respectively, according to experimental results. The formation of heterojunctions endows composite photocatalysts with higher hydrogen evolution rates compared to single-component catalysts. This is attributed to energy band bending at the interface of the heterojunction, which facilitates efficient charge separation while maintaining strong redox capabilities. High-value compounds like phenol and acetophenone were formed when the oxidation products in the post-reaction lignin model compound solution were subsequently analyzed using high-performance liquid chromatography. Additionally, a convincing mechanism for the catalytic reaction was suggested. It is expected that this study will offer a viable route for the creation of effective photocatalytic materials, high-value organic waste transformation, and sustainable hydrogen production.

## 1. Introduction

The creation of sustainable and green energy sources to replace traditional fossil fuels has become a top priority for the international scientific community due to the growing energy problem and increasing environmental degradation [1,2]. Hydrogen energy exhibits considerable development potential and several application opportunities in future energy systems as a clean energy carrier with a high energy density and zero carbon emissions [3,4,5,6]. Photocatalytic biomass reforming for hydrogen production enables the efficient integration of solar energy with biomass resources [7,8]. While oxidizing biomass model compounds (such as lignin derivatives), this technology can simultaneously achieve clean and efficient hydrogen generation, thus offering an ideal solution for addressing energy and environmental challenges [9,10].

At present, the development of highly efficient photocatalysts remains the principal barrier to the large-scale application of this technology [11,12,13]. An outstanding photocatalyst must possess robust redox capabilities to efficiently facilitate both biomass oxidation and proton reduction. Nevertheless, the present metal sulfide catalysts have poor visible light absorption capabilities and readily recombine photogenerated charge carriers. These drawbacks restrict their photocatalytic performance [14,15]. Multi-metallic sulfides have drawn much interest lately because of their exceptional visible light collecting capabilities and adjustable band structures. Among them, ZnCdS (ZCS) solid solutions exhibit both a narrow band gap and strong electron reduction capability [16,17], making them highly promising photocatalysts for hydrogen production [18]. However, single-component ZCS materials face issues such as low separation efficiency in photogenerated charge carriers and susceptibility to photo-corrosion. These problems not only reduce hydrogen production efficiency but also affect long-term stability, thereby limiting further application of the material [19,20].

In order to improve the separation efficiency of photogenerated electron–hole pairs and reduce photo-corrosion, ZCS can be integrated with other functional semiconductor materials to obtain proper band alignment and effective charge carrier separation. When exposed to solar light, a Ni_0.8_Co_0.2_/Zn_0.5_Cd_0.5_S composite photocatalyst made by Yue et al. [21] produced a maximum hydrogen generation rate of 0.21 mmol·g^−1^·h^−1^. Dai et al. [22] loaded a CoP cocatalyst onto the ZCS semiconductor, successfully constructing the CoP/ZCS heterojunction. According to their findings, the 5% CoP/ZCS heterojunction demonstrated the greatest hydrogen evolution capability, with a rate of 0.73 mmol·g^−1^·h^−1^, which is double that of Pt/ZCS and 20 times greater than that of ZCS. The close contact between the two semiconductors in the CoP/ZCS heterojunction speeds up the separation and migration of photogenerated charge carriers and lowers the surface resistance for the movement of electrons. Li et al. [23] successfully fabricated an S-type Co_9_S_8_/Zn_0.5_Cd_0.5_S heterojunction by in situ growth of ZCS nanospheres on the surface of the Co_9_S_8_ cocatalyst, which effectively suppressed the aggregation of ZCS. When the loading amount of Co_9_S_8_ reached 10%, the hydrogen production rate achieved 10.90 mmol·g^−1^·h^−1^. Although Co has been widely utilized as a metallic support in photocatalytic hydrogen evolution, research on the application of the metal oxide CoWO_4_ in photocatalytic conversion of lignocellulose is still lacking. As a narrow-bandgap transition metal oxide, CoWO_4_ exhibits excellent photoelectronic properties and catalytic activity. Its combination with ZnCdS is expected to synergistically enhance the overall photocatalytic performance.

Therefore, this study focuses on the ZnCdS/CoWO_4_ heterojunction as the research subject. Cd is firmly immobilized within the stable ZCS/CW composite matrix, thereby minimizing the potential risk of leaching during operation. To verify that the heterojunction was successfully constructed, a number of characterization techniques were used. The process was then qualitatively examined using high-performance liquid chromatography, which clarified the oxidation pathway during the photocatalytic transformation of a lignin model compound. The reaction mechanism of biomass photo-reforming for the evolution of hydrogen was further clarified by choosing sodium lignosulfonate as the substrate to measure the hydrogen production yield in the photocatalytic process. Although ZnCdS-based composite catalysts have been studied for photocatalytic hydrogen production, research focusing on the photo-reforming of biomass model compounds (such as lignin and its derivatives) using these catalysts remains scarce. This study not only aims for the efficient and clean production of hydrogen, but also explores the high-value conversion of biomass resources, thereby aligning with the current trends in sustainable development. In addition to offering theoretical underpinnings and technological references for the high-value exploitation of biomass and clean energy generation, this research offers fresh perspectives on the design and development of highly effective photocatalytic materials for hydrogen production.

## 2. Materials and Methods

### 2.1. Preparation of Photocatalysts

Preparation of Zn_0.5_Cd_0.5_S: In a beaker with 50 mL of deionized water, Zn(CH_3_COO)_2_·2H_2_O (1.10 g, 5 mmol) (Tianjin Damao Chemical Reagent Co., Ltd., Tianjin, China), Cd(CH_3_COO)_2_·2H_2_O (1.33 g, 5 mmol) (Aladdin Reagent Co., Ltd., Angeles, CA, USA), and C_2_H_5_NS (0.75 g, 10 mmol) (Kelong Chemical Reagent Co., Ltd., Chengdu, China) were added, and the mixture was agitated for 10 min at room temperature. Then, using a rubber-tipped dropper, 20 mL of NaOH solution (1 mol/L) (Tianjin Damao Chemical Reagent Co., Ltd., Tianjin, China) was added dropwise, and the mixture was constantly swirled for 30 min. Following its transfer into a high-pressure reactor, the suspension was kept at 180 °C for 24 h before naturally cooling to ambient temperature. Ultimately, the product was cleaned three times using ethanol and deionized water successively. After that, the material was dried for 12 h at 60 °C to produce yellow ZCS powder.

Preparation of CoWO_4_: Co(NO_3_)_2_·6H_2_O (1.46 g, 5 mmol) (Macklin Biochemical Co., Ltd., Shanghai, China) and NaWO_4_·2H_2_O (1.65 g, 5 mmol) (Tianjin Chemical Reagent Co., Ltd., Tianjin, China) were dissolved in 30 mL of deionized water in a beaker to form a homogeneous dispersion. After 30 min of stirring at room temperature, the mixture was moved to a high-pressure reactor and kept at 180 °C for 24 h. The final product was allowed to naturally cool to room temperature before being successively cleaned three times using ethanol and deionized water. It was then dried for 12 h at 60 °C to produce CW blue powder.

**Figure 1:**
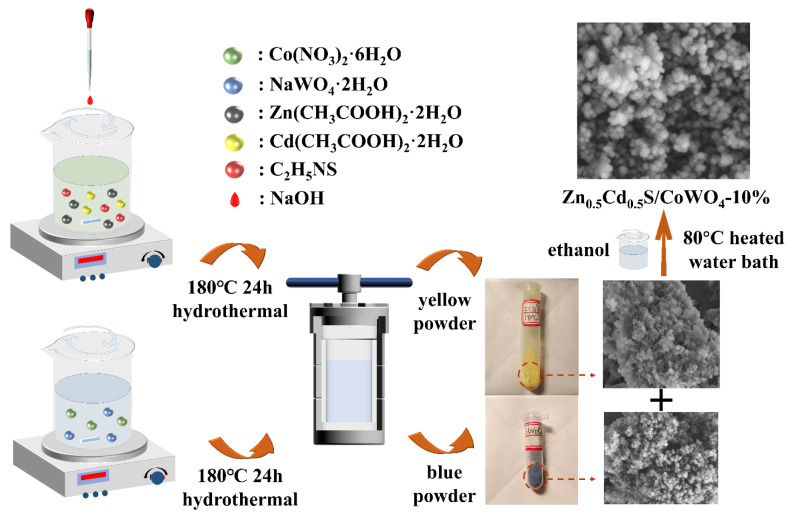
Diagram illustrating the synthesis process of the photocatalytic composite.

Preparation of Zn_0.5_Cd_0.5_S/CoWO_4_-10% Composite: After adding 0.01 g of CoWO_4_ powder and 0.1 g of Zn_0.5_Cd_0.5_S powder to 20 mL of ethanol, the mixture was thoroughly stirred. After that, the mixture was heated to 80 °C in a bath of water while being constantly stirred to ensure the solvent was thoroughly dissolved. The ZCS/CW-10% composite was then obtained by drying the resultant solid for 12 h at 60 °C in an oven. Figure 1 shows the thorough preparation procedure.

### 2.2. Characterization

A scanning electron microscope (GeminiSEM 360, Carl Zeiss AG, Jena, Germany) was used to examine the catalysts’ surface morphology, and a transmission electron microscope (Talos F200S, Thermo Fisher Scientific, Waltham, MA, USA) was used to describe their microstructure. To confirm the successful synthesis of the materials, X-ray powder diffraction (Empyrean, Malvern Panalytical, Malvern City, UK) was used to analyze the crystal phases and elemental content of the catalysts. The elemental composition, oxidation states, chemical bonding types, and electronic structure of the catalysts were among the surface chemical features that were examined using X-ray photoelectron spectroscopy (PHI 2000, Ulvac Phi Instruments Co., Ltd., Nanjing, China). X-ray powder diffraction (XRD) patterns were recorded using Cu Kα radiation (λ = 1.5406 Å) over a 2θ range of 10–80°, at a scan rate of 10° min^−1^ with a step size of 0.02°. The catalysts were exposed to nitrogen at a steady low temperature (77 K) after first undergoing degassing under vacuum for surface area and pore volume characterization (QUADRASORB SI). Following the attainment of adsorption–desorption equilibrium, the Brunauer–Emmett–Teller (BET) theory was used to compute the particular area of surface and average size of pores, as well as to evaluate changes in gas uptake and equilibrium pressure. The optical properties of the photocatalytic materials, such as absorption wavelength and range, were characterized using a UV–Vis spectrophotometer (Cary7000, Agilent Technologies, Inc., Santa Clara, CA, USA).

### 2.3. Electrochemical Measurements

Using a three-electrode electrochemical workstation (CHI760E, Shanghai Chenhua Instrument Co., Ltd., Shanghai, China) with the produced material as the working electrode, a platinum sheet as the counter electrode, and an Ag/AgCl electrode as the reference electrode, the photoelectrochemical characteristics of the catalysts were assessed. The working electrode was prepared by dispersing 5 mg of the catalyst in a mixed solvent comprising 100 μL of water, 200 μL of ethanol, and 50 μL of Nafion solution. The resulting suspension was uniformly coated onto the surface of an FTO substrate and dried at 60 °C for 10 min. An aqueous Na_2_SO_4_ solution (0.5 mol·L^−1^) was used as the electrolyte, ensuring that its volume filled at least two-thirds of the electrochemical cell, and the centerlines of the three electrodes were maintained at the same horizontal level. A 300 W xenon lamp served as the incident light source, and intermittent illumination was applied with 10 s light and dark cycles to assess the charge separation efficiency of photogenerated electron–hole pairs in the catalyst.

### 2.4. Photocatalytic Performance Evaluation

Using a lignin model compound 1-phenoxy-2-phenylethanol (Bide Pharmatech Ltd., Shanghai, China), photocatalytic hydrogen generation was performed in a 22 mL headspace vial. In particular, 10 mL of acetonitrile, 0.01 g of catalyst, and 0.01 g of the lignin model compound were added to the vial. To achieve homogeneous dispersion, the mixture was ultrasonicated for 20 min. Nitrogen gas was purged into the headspace container to expel the residual air in the upper part of the container in order to guarantee experimental precision. The reaction was then carried out in a system with a magnetic stirrer and cooling water running while being exposed to 76.8 mW of light intensity from a xenon lamp with a 400 nm long-pass filter (which only lets in visible light). To keep the system temperature at 6 °C during the reaction, a condenser was employed. A syringe was used to quantitatively remove 0.5 mL of gaseous from the headspace vial at one-hour intervals, and the gas was then injected into a Yidian-GC128 gas chromatograph (Shanghai Instrument & Electrical Analysis Instrument Co., Ltd., Shanghai, China) for analysis. Utilizing an external standard method, the amount of hydrogen generated was determined.

During the experiment, liquid samples collected from the reaction were filtered through a 0.22 μm membrane. High-performance liquid chromatography (HPLC) was then used to assess the product substance, and calibration curves were used to calculate the conversion. The HPLC analysis was performed under the following conditions: injection volume of 10 μL, mobile phase composed of acetonitrile and water (*v*/*v* 40:60 or 60:40), flow rate of 1.0 mL/min, Agilent Eclipse Zorbax C18 column (Santa Clara, CA, USA) maintained at 35 °C, and a UV detector set at 230 nm. The apparent quantum efficiency (AQY) is calculated according to the following formula: AQY (%) = (number of reactive electrons)/(number of incident photons) × 100%. This equation is employed to assess the efficiency of photocatalytic reactions by quantifying the utilization of incident light.

The photocatalytic hydrogen evolution tests were carried out in headspace vials with a total volume of roughly 22 mL using sodium lignosulfonate (Aladdin Reagent Co., Ltd., Angeles, CA, USA). Specifically, 0.01 g of the photocatalyst, 0.01 g of sodium lignosulfonate, and 10 mL of water were sequentially added to the vials. The mixture was then ultrasonicated for 20 min to ensure uniform dispersion of the components. In order to guarantee the accuracy and repeatability of the testing settings and outcomes, nitrogen was then forced into the system to eliminate any remaining air. The vials were subsequently placed in a reaction apparatus equipped with magnetic stirring and circulating cooling water. The hydrogen production procedures and conditions are consistent with those used above with lignin model substrates.

### 2.5. Quantitative Calculation

Using the external standard method, a calibration curve is established to correlate the peak area with the gas volume. Based on the ideal gas law (*PV* = *nRT*), the concentration of the generated hydrogen can be obtained, thereby establishing a corresponding relationship between the peak area and hydrogen concentration. In this equation, *p* represents pressure (taken as 101,325 Pa under standard conditions), *V* is the gas volume (in m^3^), R is the universal gas constant (8.314 J·mol^−1^·K^−1^), and *T* is the ambient temperature (typically set at 293.15 K). By substituting the measured peak area of H_2_ into the established calibration curve (peak area vs. concentration), the hydrogen concentration produced in the reaction can thus be determined.

The conversion rate of lignin model compounds in photocatalytic reactions is calculated as follows:

(1) $$Conversion\left(\%\right)=\frac{amount\;of\;substance\;reacted\;}{amount\;of\;substrate}\times 100\%$$

Here, the “amount of substance reacted” refers to the number of moles of the lignin model compound that have undergone transformation during the reaction, while the “amount of substrate” refers to the initial number of moles of the lignin model compound added to the reaction system. This formula quantifies the proportion of the substrate converted under the given photocatalytic conditions, providing a direct measure of the reaction’s efficiency.

## 3. Results and Discussion

### 3.1. Characterization of Photocatalysts

**Figure 2:**
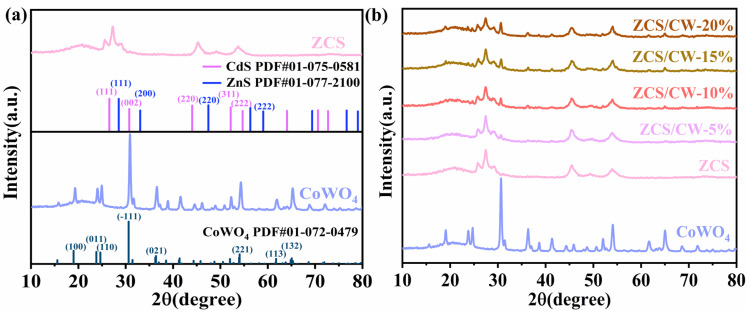
(**a**) Standard card patterns of ZCS and CW, (**b**) XRD patterns of samples.

Figure 2 displays the X-ray diffraction (XRD) profiles for ZCS, CW, and the ZCS/CW-10% composite catalysts. As observed in Figure 2a, the diffraction peaks for ZCS are evidently shifted in comparison to the standard patterns of ZnS and CdS, indicating changes in the crystal lattice. The main peaks of the ZCS composite correspond to the (111), (002), (220), and (311) planes of CdS, as well as the (111), (200), (220), and (222) planes of ZnS, signifying the successful formation of the ZCS/CW hybrid material. The crystal structure of CW matches reference PDF#01-072-0479 [24], displaying diffraction peaks at 18.99°, 23.81°, 24.65°, 30.63°, 36.41°, 54.04°, 61.75°, and 65.05°, which may be assigned to the (100), (011), (110), (–111), (021), (221), (113), and (132) planes, respectively. From Figure 2b, it is clear that the ZCS/CW sample exhibits pronounced peaks at 30.63°, 36.41°, and 65.05°, associated with the (–111), (021), and (132) planes of CW, respectively. Moreover, the intensities of these characteristic reflections grow as the CW content increases. Both the ZCS and CW components can be clearly identified from the composite’s diffraction pattern, confirming the coexistence of both phases in the final product. This indicates that the heterojunction has been successfully synthesized, and that the fundamental crystal structures of both materials are well preserved during the combination process [25].

**Figure 3:**
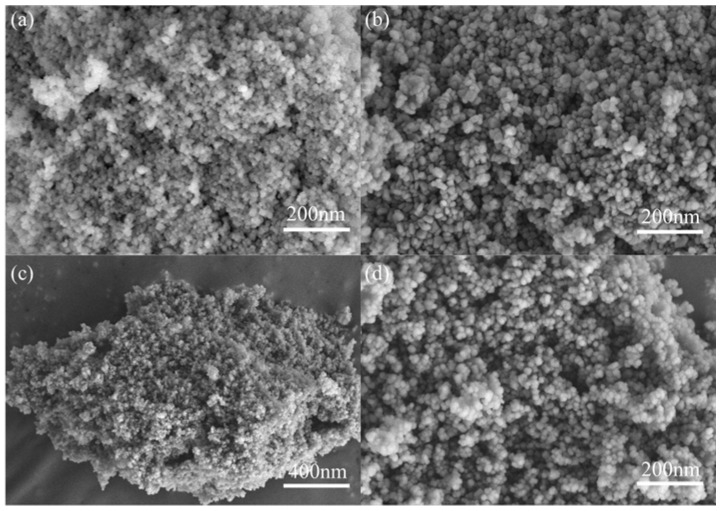
SEM images of (**a**) ZnCdS, (**b**) CoWO_4_, (**c**,**d**) SEM images of ZCS/CW-10%.

The morphological characteristics of composites were analyzed by scanning electron microscopy (SEM), as shown in Figure 3a–d. Figure 3a presents the SEM image of ZCS, where it can be observed that the material exhibits a highly aggregated nanoparticle morphology. The particle size distribution is relatively uniform, and the nanoparticles are interconnected, forming a porous network structure. Such porosity contributes to an increased specific surface area, thereby providing more active sites for the adsorption and transformation of reactants during the photocatalytic process. Figure 3b shows the surface morphology of CW. Compared with ZCS, CW exhibits a rougher particle morphology and relatively larger particle size, with a distinct blocky or nearly spherical distribution. The interfaces between the particles are tighter, which is beneficial for the efficient transport of charge carriers. As shown in Figure 3c,d, the ZCS/CW-10% composite catalyst exhibits mutually encapsulated particles, forming loose and uniformly distributed agglomerates. Compared with the two individual component materials, the composite presents a more loosely arranged particle structure. The pronounced agglomeration indicates that the hierarchical structure of the material has been effectively modulated through the composite process. In addition to increasing the material’s specific surface area, the abundant nanoscale porous structure offers more channels for reactant molecule diffusion and photocatalytic reaction progression, which enhances the separation efficiency of photogenerated charge carriers.

**Figure 4:**
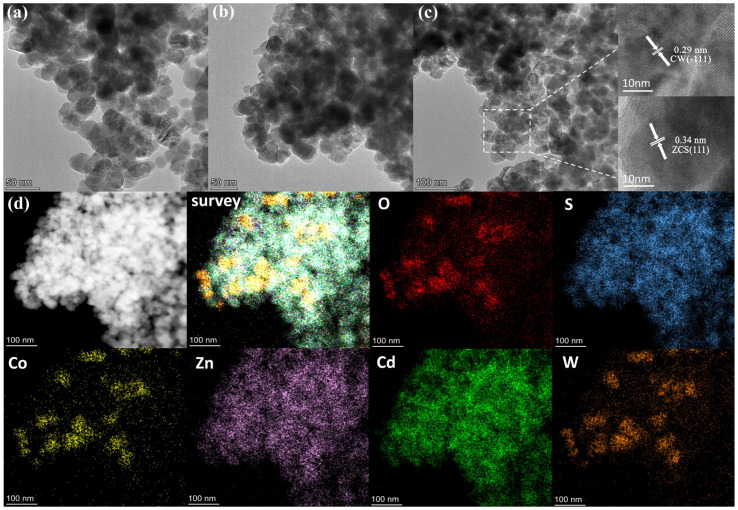
(**a**–**c**) TEM images of composite materials, (**d**) EDX elemental mapping.

To further investigate the microstructure of ZCS/CW-10%, transmission electron microscopy (TEM) analysis was performed. As shown in Figure 4a–c, the composite material is mainly composed of uniformly sized and well-dispersed nanoparticles with diameters of 20–30 nm, which are interconnected to form an aggregated network structure. Additional analysis of the high-resolution TEM (HR-TEM) pictures shows clear lattice fringes with 0.34 nm and 0.29 nm spacing, which represent the (111) plane of ZCS and the (–111) plane of CW, respectively. These features provide strong evidence for the coexistence and intimate combination of both phases at the nanoscale, and demonstrate the formation of hetero-structured interfaces. These interfaces help to improve the efficiency of photocatalytic reactions by improving the division of electron–hole pairs.

The energy-dispersive X-ray spectroscopy (EDS) data shown in Figure 4d provide detailed insights into the elemental spatial distribution within the ZCS/CW-10% composite. The comprehensive elemental mapping indicates that all constituent elements are homogeneously dispersed across the entire composite, with no observable evidence of phase separation or localized agglomeration. This even distribution of elements strongly substantiates the effective synthesis of the ZnCdS/CoWO_4_-10% composite catalyst, suggesting that ZnCdS and CoWO_4_ components are intimately integrated at the atomic scale, thereby achieving a structurally uniform composite material [26]. The individual elemental mapping images clearly demonstrate that S, Zn, and Cd are uniformly dispersed among the nanoparticles. Co, W, and O elements exhibit localized aggregation or dispersion within the pore structure of the ZnCdS material. S, Zn, and Cd serve as characteristic elements of the ZnCdS phase, while O, Co, and W are primarily associated with the CoWO_4_ phase. These results further confirm the effective nanoscale interpenetration of each component, providing a robust basis for the synergistic effects and superior photoelectronic properties of the composite material.

**Figure 5:**
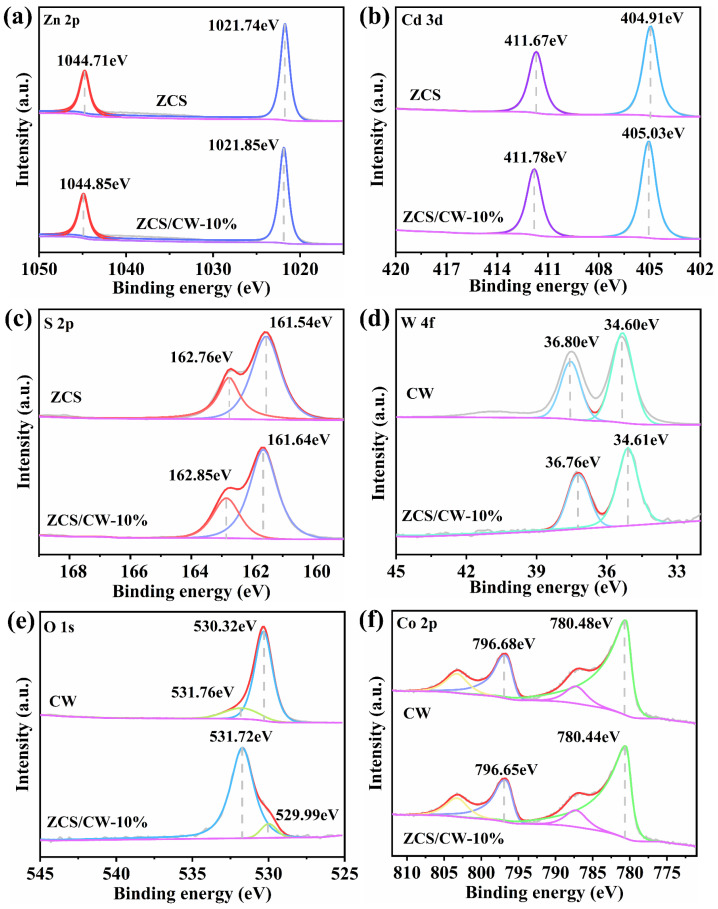
Fine spectrum of (**a**) Zn 2p, (**b**) Cd 3d, (**c**) S 2p, (**d**) W 4f, (**e**) O 1s, (**f**) Co 2p.

X-Ray photoelectron spectroscopy (XPS) was used to evaluate the elemental valence states and surface chemical compositions of ZCS, CW, and ZCS/CW-10%, as illustrated in Figure 5. The Zn 2p_3/2_ and Zn 2p_1/2_ orbitals are represented by two prominent peaks for Zn 2p in the ZCS XPS spectra, which are located at 1021.74 eV and 1044.71 eV, respectively, as seen in Figure 5a. The peak positions that have been detected verify that Zn is present in the ZCS material in the +2 oxidation state. As can be seen in Figure 5b, the Cd 3d XPS spectrum shows a distinct doublet. The Cd 3d_5/2_ and Cd 3d_3/2_ orbitals are responsible for the binding energies at 404.91 eV and 411.67 eV, respectively. These findings show that the catalyst also contains Cd in the +2 oxidation state [27]. The distinctive peaks located at 161.54 eV and 162.76 eV in Figure 5c are attributed to S 2p_3/2_ and S 2p_1/2_, respectively. Furthermore, compared with pure ZCS, the binding energies of the Zn 2p, Cd 3d, and S 2p orbitals are shifted to higher values in the ZCS/CW-10% sample. This indicates that the introduction of CW induces strong interactions between the components. The observed shift suggests that electrons transfer from ZCS to CW when the two materials come into contact. The W 4f_7/2_ and W 4f_5/2_ peaks are situated at 34.60 eV and 36.80 eV, respectively, as illustrated in Figure 5d. Figure 5f demonstrates that the Co 2p peaks in CW appear at 780.48 eV and 796.68 eV, corresponding to the Co 2p_3/2_ and Co 2p_1/2_ orbitals, respectively, with clear satellite peaks observed. Under illumination, the binding energies of Zn, Cd, and S in the ZCS/CW-10% sample are shifted to lower values, whereas those of W and Co are shifted to higher values. This result shows that photogenerated electrons move from ZCS to the CW surface over the S-type heterojunction when exposed to light. Subsequently, these electrons participate in the proton reduction reaction, resulting in the generation of hydrogen [28]. The effectiveness of photogenerated electron and hole separation is greatly increased by the development of the S-type heterojunction. As a result, more e^−^ are available to participate in the reduction of h^+^, thereby accelerating the hydrogen evolution reaction.

**Figure 6:**
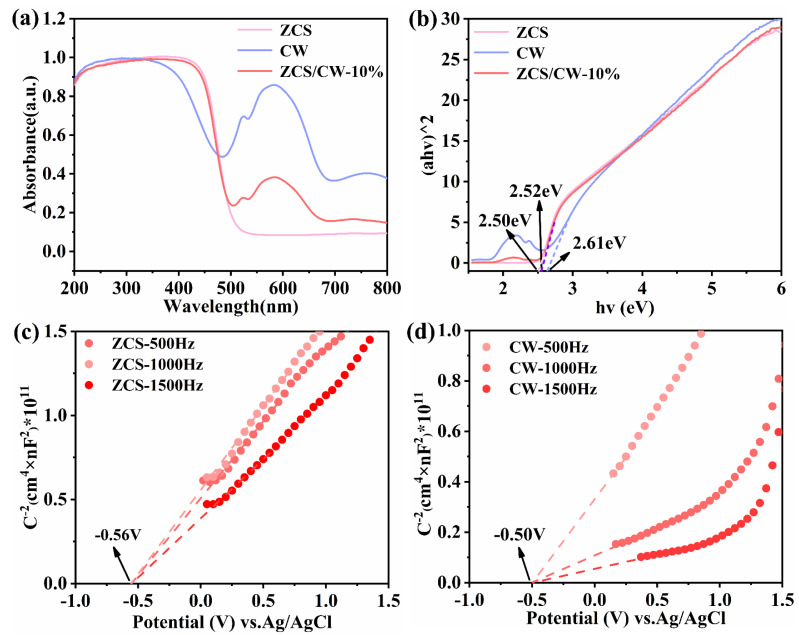
(**a**) UV–Vis absorption spectra, (**b**) the relationship between (αhυ)^2^ and (hυ), (**c**,**d**) Mott Schottky plots of ZCS and CW.

UV–Vis diffuse reflectance spectroscopy (UV–Vis DRS) was used to examine the optical absorption characteristics of materials. As shown in Figure 6a, according to the UV–Vis data, ZCS exhibits an absorption band centered at 500 nm. Due to inter-band electronic transitions, CW exhibits a broad absorption range spanning both the visible and ultraviolet spectra, with a distinct absorption peak at 580 nm. This observation is consistent with the XRD results. Compared to ZCS, ZCS/CW-10% shows a slight blue shift in the absorption edge, along with an increased absorption intensity in the 200–500 nm range. This suggests that the composite can utilize solar energy more effectively. Based on the relationship between (αhν)^2^ and hν, the estimated band gaps of ZCS, CW, and ZCS/CW-10% are 2.50 eV, 2.61 eV, and 2.52 eV, respectively, as shown in Figure 6b. It is evident that the incorporation of CW has little effect on the band position of ZCS. To further clarify the catalytic oxidation mechanism, it is essential to determine the conduction and valence band positions of the catalysts. Therefore, Mott–Schottky measurements were conducted for both ZCS and CW. As illustrated in Figure 6c,d, the *x*-axis intercept for ZCS is measured at –0.56 eV and CW is measured at –0.50 eV, respectively. The more negative flat-band potential of ZCS indicates an enhanced reduction capability, facilitating the participation of photogenerated electrons in reduction processes. The conduction band positions of ZCS and CW are −0.66 eV and −0.60 eV versus the normal hydrogen electrode (NHE), respectively, due to the use of Ag/AgCl as the reference electrode in the experiment. The valence band locations of ZnCdS and CoWO_4_ are determined to be 1.84 eV and 2.01 eV, correspondingly, when combined with their respective band gaps.

**Figure 7:**
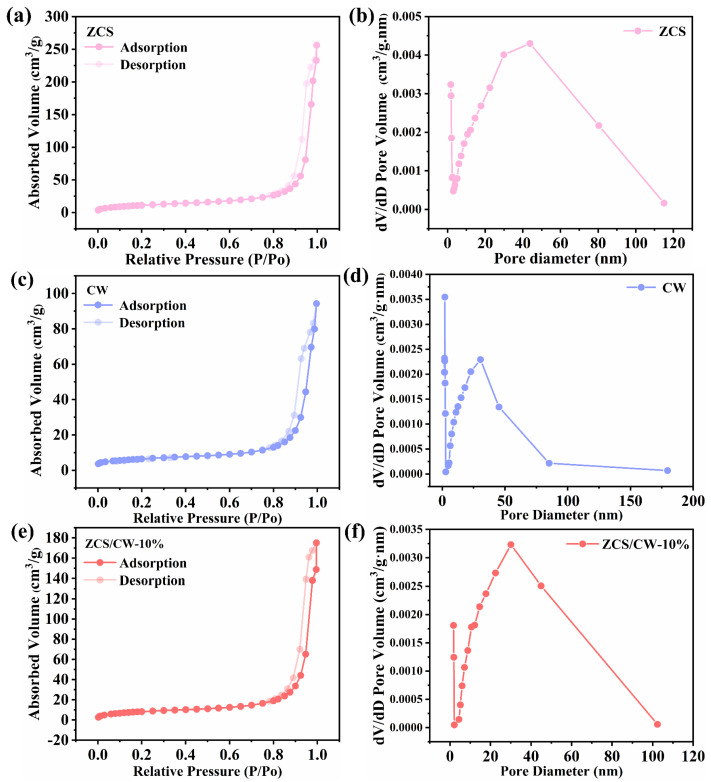
(**a**,**b**) ZCS, (**c**,**d**) CW, (**e**,**f**) ZCS/CW-10% nitrogen adsorption–desorption isotherms and pore size distribution curves.

The samples were subjected to nitrogen adsorption–desorption (BET) experiments to examine the specific surface area and pore structure of the catalysts. As shown in Figure 7a–f, all materials exhibited type IV adsorption isotherms, which are characteristic features of mesoporous materials [29]. BET results indicate that ZCS possesses the largest specific surface area (40.27 m^2^/g), whereas CW shows a relatively lower value (21.53 m^2^/g). The specific surface area of ZCS/CW-10% is 29.16 m^2^/g, lying between those of ZCS and CW. According to Table 1, the pore size of the composite ZCS/CW-10% is 29.27 nm, which is also intermediate between ZCS (30.98 nm) and CW (24.42 nm). These results indicate that the integration of ZCS and CW effectively optimizes the pore structure of the composite catalyst. Compared with the individual components ZnCdS and CoWO_4_, the mesoporous structure of the composite material ZCS/CW-10% significantly enhances light absorption and facilitates the mass transfer and diffusion of reactants. Therefore, catalytic activity should not be judged solely by specific surface area, but should also take into account both surface area and pore structure.

**Figure 8:**
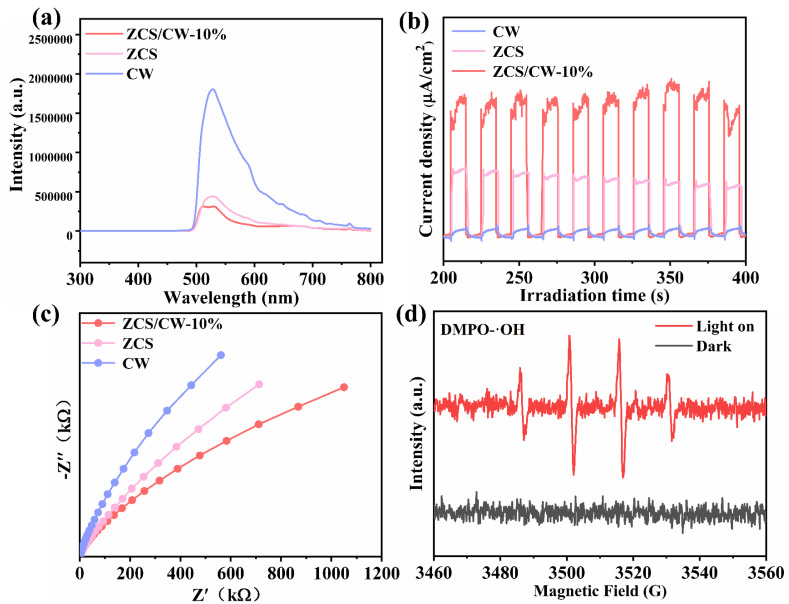
CW, ZCS, ZCS/CW-10% of (**a**) Steady-state fluorescence spectra, (**b**) Transient photocurrent response, (**c**) Electrochemical impedance, (**d**) ESR test of ZCS/CW-10%.

Photoelectrochemical experiments were carried out for photocatalysts in order to examine the migration, separation, and recombination processes of photogenerated electrons in the photocatalysts. As shown in Figure 8a, the steady-state photoluminescence (PL) spectra reveal that ZCS/CW-10% exhibits the lowest PL intensity, while CW displays the highest. Figure 8b presents the photocurrent responses of the three materials under illumination. All samples show a photocurrent response, with CW exhibiting the weakest, which is consistent with the PL results. ZCS displays a moderate response, whereas the composite ZCS/CW-10% shows the highest photocurrent intensity. This suggests that, after forming a heterojunction with CW, an effective internal electric field is generated in ZCS/CW-10%, which facilitates the directional migration of photogenerated charge carriers. The ZnCdS/CoWO_4_-10% has the lowest Nyquist circle following compositing, as illustrated in Figure 8c, suggesting that the addition of CW greatly improves the transmission efficiency of charges produced by photons and inhibits charge carrier recombination [30]. These results collectively demonstrate that the composite possesses the most efficient photogenerated carrier separation ability, which is consistent with its outstanding hydrogen production performance.

As shown in Figure 8d, the electron spin resonance (ESR) spectrum of ZCS/CW-10% exhibits no detectable signal under dark conditions. Upon light irradiation, a distinct signal emerges, indicating the generation of highly oxidative hydroxyl radicals under photoexcitation. The signal shows a relative intensity ratio of 1:2:2:1 [31]. The standard potential for the conversion of OH^−^ to ·OH is E_0_(OH^−^/·OH) = 2.40 eV. Since the potential of the catalyst is insufficient to drive this transformation, OH^−^ cannot be directly oxidized to ·OH. Therefore, holes act as the sole oxidizing species in the reaction. This enables lignocellulose to play a dual role: it serves as a sacrificial agent to consume holes and suppress the recombination of charge carriers, while also being oxidized to produce small molecular compounds. The ·OH radicals detected by ESR arise from the following process: upon electron–hole separation, conduction band electrons react with dissolved oxygen to generate superoxide radicals (·O_2_): e^−^ + O_2_ →·O_2_. Some of these superoxide radicals further react with water to form hydroxyl radicals:·O_2_ + 2H_2_O → 4·OH. Based on the above analysis, the presence of holes can be substantiated.

### 3.2. Synergistic Photocatalytic Hydrogen Evolution Performance Analysis of Lignin Model Compounds

**Figure 9:**
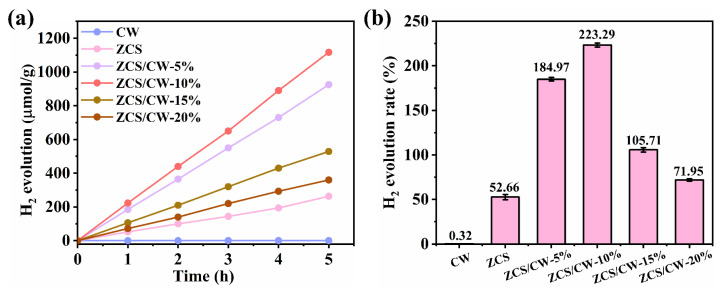
(**a**) Hydrogen production rate graph, (**b**) Comparison of hydrogen production rates (the error bar represents the standard deviation, *p* < 0.05).

Firstly, PP-ol (2-phenoxy-1-phenylethanol), a well-characterized lignin model compound with a relatively small molecular weight, was chosen as the substrate to examine the photocatalyst’s synergistic hydrogen production performance during the photo-reforming of lignin model compounds. Figure 9a presents the hydrogen evolution activities of catalysts. As shown, ZCS exhibits relatively low hydrogen production performance. Combined with Figure 9b, it can be seen that its hydrogen evolution rate is only 52.66 μmol·g^−1^·h^−1^. The performance of photocatalytic hydrogen evolution is improved by the addition of CW. The hydrogen generation rate steadily rises when CW loading is gradually increased, reaching a peak of 223.29 μmol·g^−1^·h^−1^ at a 10% CW loading percentage. But when the CW content is increased further, the hydrogen evolution rate significantly decreases. Therefore, ZCS/CW-10% is identified as the optimal composite ratio for photocatalytic activity.

**Figure 10:**
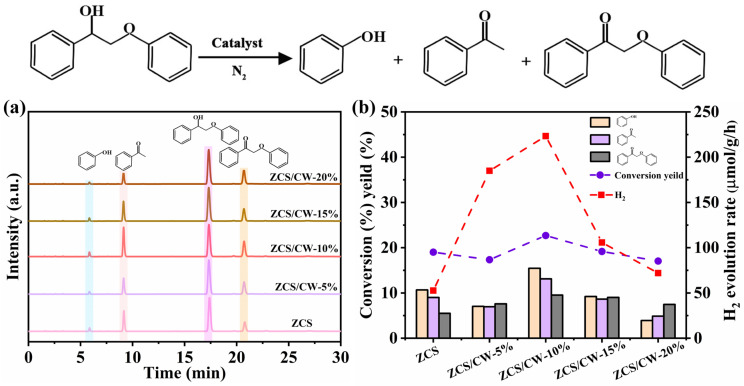
(**a**) HPLC spectra of the solution under different CW loadings, (**b**) Conversion rate and hydrogen production under different CW loadings.

To elucidate the oxidation pathway, high-performance liquid chromatography (HPLC) was employed to analyze the reaction solution. When a well-defined lignin model compound is used as a sacrificial agent, the oxidation pathway of the model compound and its correlation with hydrogen production can be evaluated by analyzing the products generated through liquid chromatography. As shown in Figure 10a,b, four compounds are detected during the ZCS-catalyzed oxidation of the model compound: phenol, acetophenone, intermediate products, and incompletely oxidized model compound. After CW is loaded onto the surface of ZCS, the yield of intermediate products increases significantly with increasing CW content, and the amounts of phenol and acetophenone also increase. When the CW loading reaches 10%, both the conversion rate and hydrogen production reach their maximum values. However, when the CW loading exceeds 10%, both the conversion rate and hydrogen production show a decreasing trend. The production of H_2_ matches up with that of the intermediate product, as illustrated in Figure 10b. This implies that the creation of a heterojunction between ZCS and CW enhances the oxidation capability of the photocatalyst and facilitates the initial transformation of the model compound, leading to the formation of additional intermediates. Specifically, the C_α_H-OH group is oxidized to C_α_=O, and the released hydrogen ion are reduced by electrons to form H_2_. Therefore, the formation of intermediate products facilitates the hydrogen evolution reaction. The relationship between conversion rate and hydrogen production is shown in Table 2.

**Figure 11:**
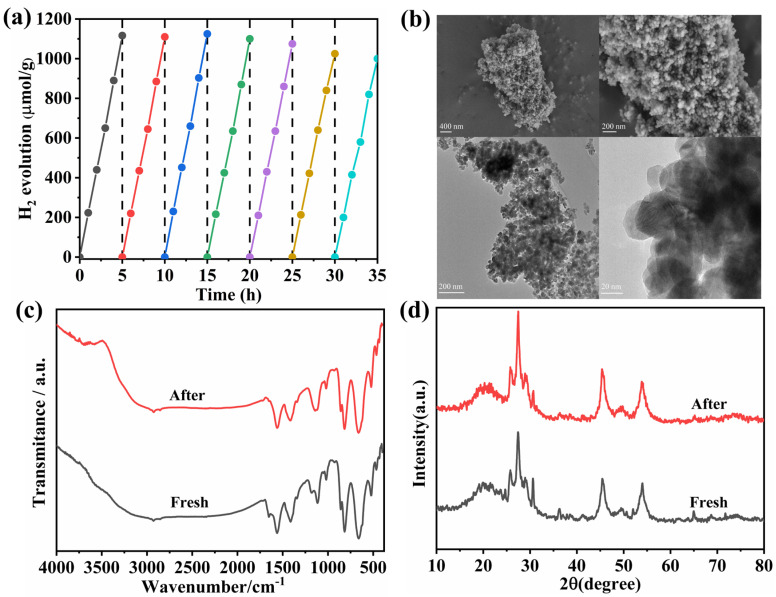
(**a**) Photocatalytic hydrogen production cycling experiment conducted on the ZCS/CW-10% sample, (**b**) SEM and TEM profile of ZCS/CW-10% material after the reaction, (**c**) FT-IR pattern and (**d**) XRD pattern of the fresh and recycled photocatalyst.

To evaluate the stability of the catalyst, seven consecutive hydrogen evolution cycles were conducted using the composite material. The hydrogen production remained consistently around 1116 μmol·g^−1^ within 5 h for each cycle, indicating excellent catalytic stability. As seen in Figure 11, the catalyst was investigated by TEM, FT-IR, and XRD following the cycle tests. After cycling, the CW nanoparticles remained tightly encapsulated within the ZCS nanoclusters, which was consistent with the TEM results observed before cycling. While the XRD patterns revealed no discernible change in the crystal phase, the infrared diffraction peaks only slightly altered before and after the reaction. These results collectively demonstrated that the catalyst possessed robust structural and chemical stability.

### 3.3. Synergistic Photocatalytic Hydrogen Evolution Performance Analysis of Sodium Lignosulfonate

**Figure 12:**
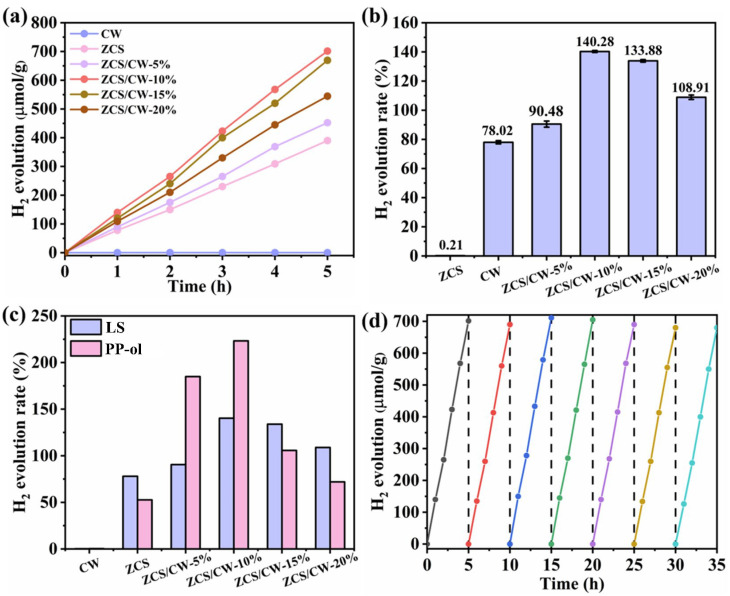
(**a**) Hydrogen production rate graph, (**b**) Comparison of hydrogen production rates, (**c**) Comparison of hydrogen production rates of sodium sulfonate and model compounds for catalysts, (**d**) Photocatalytic hydrogen production cycle experiment conducted on the ZnCdS/CoWO_4_-10% sample (the error bar in the (**b**) represents the standard deviation, *p* < 0.05).

Furthermore, to explore the potential application of the catalyst in biomass photo-reforming for hydrogen production, sodium lignosulfonate (C_20_H_24_Na_2_O_10_S_2_), a byproduct extracted during the bamboo pulping process, was selected as the substrate for the reaction. Sodium lignosulfonate is a brown powder with a higher molecular weight than the model compounds and exhibits excellent water dispersibility. Figure 12a presents the hydrogen evolution rates of catalysts using sodium lignosulfonate as the substrate. It can be observed that ZnCdS/CoWO_4_-10% exhibits the highest hydrogen generation rate, which is significantly greater than those of ZCS and CW. According to Figure 12b, CW shows negligible photocatalytic hydrogen production activity, with almost no hydrogen detected within five hours. Additionally, ZCS exhibits a low rate of evolution of hydrogen activity (78.02 μmol·g^−1^·h^−1^). When ZCS and CW are combined, the production of hydrogen grows with increasing CW loading. When the CW content reaches 10%, hydrogen production achieves its maximum value. However, with further increases in CW content beyond 10%, a significant decrease in hydrogen production is observed. One possible reason is that excessive CW loading may cover some of the active sites on ZCS, thereby hindering light absorption by ZCS. Another reason could be that CW acts as a recombination center for photogenerated electrons and holes. When too much CW is present, it tends to capture these charge carriers after their transfer to ZCS, resulting in recombination rather than effective transfer. The strong interfacial contact formed between ZCS and CW is responsible for the increased light absorption shown in the composite material. This well-designed interface makes it possible for electrons to move to the outermost layer of the composite photocatalyst and participate in the development of hydrogen, which significantly facilitates the separation of photogenerated pairs of electrons and holes under light. Figure 12c compares the hydrogen production amounts when using sodium lignosulfonate and a lignin model compound as sacrificial agents. The catalyst has excellent stability because both show a trend of initially increasing and then decreasing hydrogen production, with both reaching their maximum when the CW loading reached 10%. In general, the lignin model compound produces more hydrogen than sodium lignosulfonate. In particular, with the ZnCdS/CoWO_4_-10% catalyst, the hydrogen production rate for the lignin model compound reaches 223.30 μmol·g^−1^·h^−1^, while that for sodium lignosulfonate is only 140.28 μmol·g^−1^·h^−1^. This difference may be attributed to the lower molecular weight of the lignin model compound, which allows it to be more easily oxidized and decomposed into smaller molecules. In contrast, sodium lignosulfonate has a higher molecular weight and a more complex oxidation pathway, making it more difficult to break down into smaller molecules. Under irradiation with wavelengths below 400 nm, and using 10 mg sodium lignosulfonate as the substrate, the 10 mg ZCS/CW-10% heterojunction exhibited an apparent quantum efficiency (AQY) of 1.1% for water photocatalysis. AQY serves as a key metric for evaluating the effective utilization of incident photons by the material, reflecting the proportion of absorbed photon energy converted into active charge carriers that drive chemical reactions. This result further corroborates the excellent photocatalytic hydrogen evolution performance of the heterojunction under visible light, demonstrating its high efficiency in harnessing photonic energy for hydrogen generation. The hydrogen evolution activity of the ZCS/CW-10% composite catalyst is evaluated by cycling tests, as shown in Figure 12d. After seven cycles, the hydrogen production remains at approximately 701 μmol·g^−1^ within five hours, indicating that the catalyst maintains good hydrogen evolution activity. The hydrogen production results using sodium lignosulfonate as the substrate are presented in Table 3.

### 3.4. Analysis of the Mechanism of Photocatalytic Hydrogen Productions

**Figure 13:**
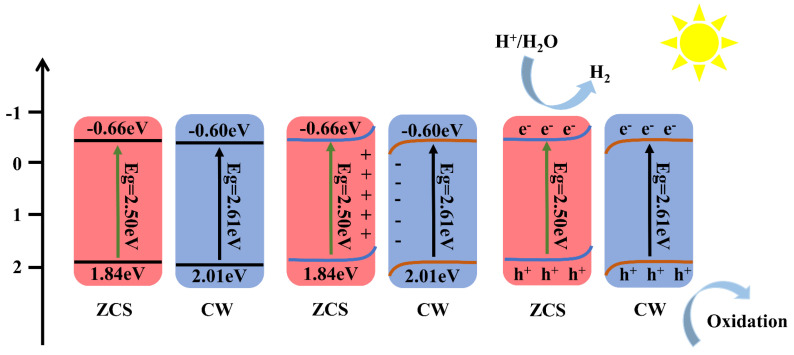
Charge Transfer at the Interface Between ZCS and CW.

A possible process for the photocatalytic reaction of the heterojunction is suggested based on the properties and analyses above, as shown in Figure 13. When two different Fermi-level materials come into connection, electrons naturally move from the higher-fermi-level material to the lower-fermi-level material. Until the Fermi levels at the interface equilibrate, this charge transfer keeps happening. Electrons transfer from ZnCdS to CoWO_4_ across their interface until equilibrium is reached, driven by the difference in their Fermi levels. Near the interface, this charge redistribution causes an interfacial electric field to develop, which causes downward band bending in CoWO_4_ and upward band bending in ZnCdS. The resulting built-in electric field effectively suppresses the recombination of photogenerated charge carriers by facilitating their efficient spatial separation and directional migration. These findings support the ZnCdS/CoWO_4_-10% catalyst’s S-type heterojunction structure, which improves the catalyst’s overall charge transfer efficiency and photocatalytic activity.

**Figure 14:**
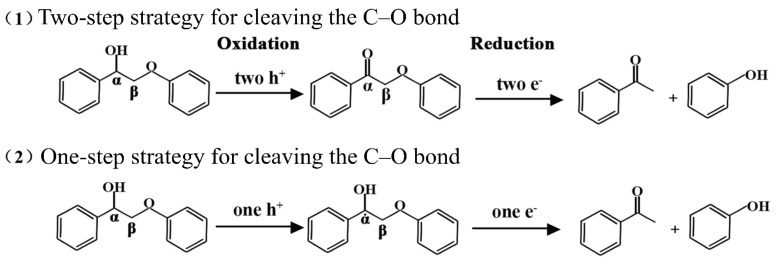
Method for the cleavage of C–O bonds in lignin model compounds under light irradiation.

In this study, C–O bond cleavage is the main mechanism by which lignin model compounds undergo photocatalytic conversion for the production of hydrogen. The breaking of the C–O bond can proceed via either a two-step or a one-step mechanism [32]. The detailed pathways of these cleavage mechanisms are illustrated in Figure 14. In the two-step mechanism, the reaction first involves the oxidation of C_α_H-OH to C_α_=O. Subsequently, the C_β_–O bond is cleaved directly or indirectly by electrons generated from the photocatalyst. Studies have shown that when the hydrogen atom in C_α_H-OH is removed and oxidized to form C_α_=O, the bond energies of the adjacent chemical bonds are altered [33]. In particular, the C_β_–O bond energy is significantly weakened, thereby facilitating its cleavage. one-step photocatalytic oxidative breaking of the C–O bond has a different process. Photogenerated holes first oxidize the substrate, leading to dehydrogenation and the formation of H^+^ and C_α_ radical. Subsequently, photogenerated electrons are transferred directly to the C_α_ radical, resulting in the cleavage of the β-O-4 structure and the production of phenol and acetophenone. It is clear that, when exposed to visible light, the primary distinction between the two depolymerization pathways of lignin model compounds is the variation in the reaction intermediates. The one-step method generates a C_α_· radical, while the two-step method produces a C_α_-O· radical, which can be further converted into a C_α_=O ketone intermediate.

**Figure 15:**
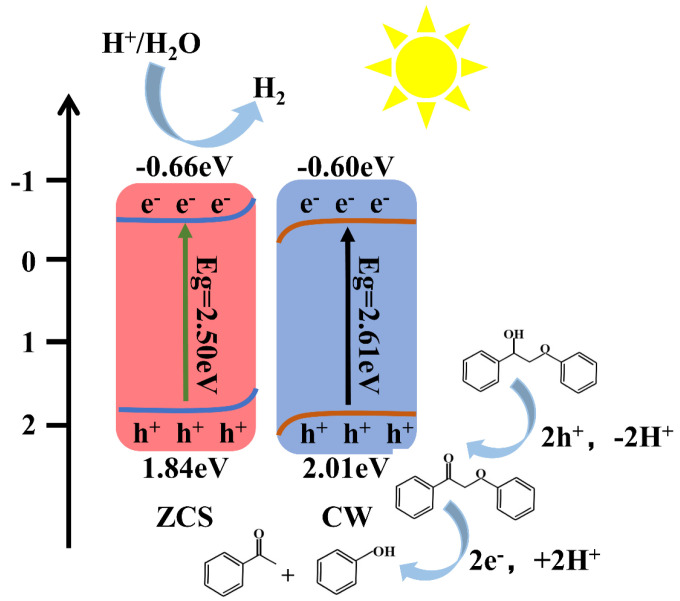
Mechanism of hydrogen production from photocatalytic reforming of lignin model compounds by ZCS/CW-10%.

Figure 15 illustrates the depolymerization process of 2-phenoxy-1-phenylethanol. Based on a comprehensive evaluation of depolymerization behavior and hydrogen evolution, the two-step mechanism is more suitable for explaining the synergistic photocatalytic hydrogen production from lignin model compounds over ZCS/CW-10%. Upon oxidation of C_α_H-OH to C_α_=O, two hydrogen ions are released, which are subsequently reduced by electrons on the ZCS catalyst to produce hydrogen gas. However, in the second step, the formation of phenol and acetophenone requires the consumption of two hydrogen ions, thereby decreasing hydrogen generation. This indicates a competitive relationship between the two processes. Therefore, the formation of the intermediate plays a crucial role in determining hydrogen production: the greater the amount of ketone intermediate formed, the higher the hydrogen yield.

## 4. Conclusions

In this study, ZCS/CW heterojunction composite photocatalysts were successfully synthesized by in situ deposition of calcium sulfate nanoparticles onto ZCS nanoclusters via hydrothermal and water bath methods. The effective formation of the ZCS/CW heterojunction structure was confirmed by TEM, XRD, and XPS characterization. Future research will focus on enhancing the stability and scalability of the catalyst, as well as investigating its performance with real biomass feedstocks under practical conditions. The main conclusions of this study are as follows:

1. The construction of the heterojunction improved charge separation, enhanced redox capability, and optimized interfacial contact between ZCS and CW, thereby significantly boosting the photocatalytic hydrogen evolution activity from lignocellulose.
2. When the CW content reached 10%, the ZCS/CW-10% photocatalyst exhibited the highest hydrogen evolution rate: 223.30 μmol·g^−1^·h^−1^ using lignin model compounds and 140.28 μmol·g^−1^·h^−1^ using sodium lignosulfonate, which was 1.6 times higher than that for sodium lignosulfonate. The superior performance of lignin model compounds is attributed to their lower molecular weight and greater susceptibility to oxidative degradation compared to the more oxidation-resistant sodium lignosulfonate.
3. High-performance liquid chromatography (HPLC) analysis revealed that the photocatalytic oxidation of lignin model compounds led to the formation of phenol, acetophenone, and an intermediate compound, with hydrogen evolution being closely related to the concentration of the intermediate.
4. ESR experiments further identified the active oxygen species involved in the process, providing deeper insight into the mechanism of photocatalytic hydrogen evolution.

## Figures and Tables

**Table 1:** Specific surface area and pore size characteristics of ZCS,CW,ZCS/CW-10%.

Sample Name	Surface Area (m^2^/g)	Pore Volume (m^3^/g)	Bore Diameter (nm)
ZCS	40.2655	0.3119	30.9891
CW	21.5298	0.1315	24.4268
ZCS/CW-10%	29.1617	0.2234	29.2744

**Table 2:** Hydrogen evolution yield via photocatalytic conversion of lignin model compounds catalyzed by ZCS/CW-x%.

	Catalyst	Reaction Time (h)	Conversion Rate (%)	Hydrogen Production per Unit Time (μmol/g/h)
1	CW	5	0	0.32
2	ZCS	5	19.02	52.66
3	ZCS/CW-5%	5	17.35	184.97
4	ZCS/CW-10%	5	22.69	223.29
5	ZCS/CW-15%	5	19.17	105.71
6	ZCS/CW-20%	5	17.05	71.95

**Table 3:** Hydrogen production yield from sodium lignosulfonate photocatalysis by ZCS/CW-x%.

	Catalyst	Reaction Time (h)	Hydrogen Production per Unit Time (μmol/g/h)
1	CW	5	0.21
2	ZCS	5	78.02
3	ZCS/CW-5%	5	90.48
4	ZCS/CW-10%	5	140.28
5	ZCS/CW-15%	5	133.88
6	ZCS/CW-20%	5	108.91

## Data Availability

The original contributions presented in this study are included in the article. Further inquiries can be directed to the corresponding author.
